# Comprehensive web-based platform for advanced PCR design, genotyping, synthetic biology, molecular diagnostics, and sequence analysis

**DOI:** 10.1016/j.omtn.2025.102716

**Published:** 2025-09-12

**Authors:** Ruslan Kalendar

**Affiliations:** 1 Helsinki Institute of Life Science (HiLIFE), University of Helsinki, Biocentre 3, P.O. Box 65, Viikinkaari 1, 00014 Helsinki, Finland; 2 National Laboratory Astana, Nazarbayev University, 53 Kabanbay batyr Ave., Astana 010000, Kazakhstan

**Keywords:** MT: bioinformatics, PCR primer and probe design, loop-mediated isothermal amplification, LAMP, Kompetitive allele-specific PCR genotyping, KASP, Gibson assembly, *in silico* PCR, primer analysis, repeat identification

## Abstract

We present a comprehensive web-based platform that integrates a suite of molecular biology tools tailored for PCR-based applications. Key features include custom multiplex tiling PCR panel design for amplicon sequencing, loop-mediated isothermal amplification (LAMP), allele-specific PCR genotyping assay development, Gibson assembly, oligonucleotide analysis and design, and the *de novo* identification, classification, and visualization of repetitive sequences. The platform supports a wide range of PCR primer design tasks, including standard, multiplex, reverse, long-range, quantitative fluorescence (TaqMan and MGB probe), and bisulfite PCR. *In silico* PCR analysis ensures primer and probe specificity across diverse applications such as gene discovery via homology analysis, molecular diagnostics, DNA profiling, and repeat sequence identification. The multiplex tiling PCR tool is optimized for both next-generation and third-generation sequencing technologies. LAMP primer design includes support for loop primers that target eight distinct regions of the DNA template. The allele-specific PCR module enables genotyping of single nucleotide polymorphisms, insertions/deletions, multi-nucleotide variants, and haplotypes at specific loci. Additionally, the platform facilitates genome-wide identification, masking, and clustering of repetitive elements, including interspersed repeats and low-complexity sequences such as simple sequence repeats and telomeres. All tools are freely accessible at https://primerdigital.com/tools/.

## Graphical abstract

**Figure:**
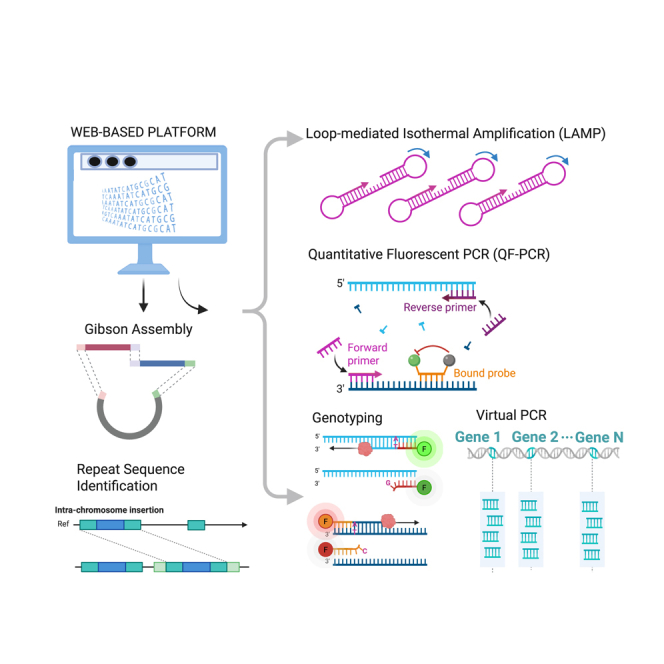

## Teaser

A multifunctional web-based platform that enables advanced PCR primer design, LAMP and genotyping assay development, *in silico* validation, and *de novo* identification of genomic repeats, providing a unified resource for molecular diagnostics and sequence analysis.

## Introduction

Contemporary molecular biology research requires increasingly demanding sophisticated computational tools capable of handling complex experimental designs and high-throughput data analysis. Although numerous algorithmic solutions exist for individual applications, the field lacks integrated platforms that efficiently combine multiple functionalities while maintaining high accuracy and computational efficiency. Despite the availability of several online solutions, there is a growing need for integrated, open, and universal platforms that can combine multiple functionalities.^1^^,^^2^^,^^3^^,^^4^

PCR is fundamental in molecular biology and is the most important practical molecular technique in research laboratories. Nucleic acid amplification assays are an essential class of specific target-sequence detection methods in contemporary biology and have a diverse range of applications, including the diagnosis of inherited diseases, human and microorganism identification, genotyping, and DNA sequencing. Numerous isothermal techniques that do not rely on thermocycling to drive the amplification reactions have been developed. For example, loop-mediated isothermal amplification (LAMP)^5^ is an isothermal nucleic acid amplification technique that does not require thermocycling. LAMP is a highly sensitive, specific, and rapid DNA amplification technique with significantly advanced molecular biology research and clinical diagnostics.

The effectiveness of DNA amplification methods largely depends on the identification of unique primer sequences with a high PCR efficiency. Primer design is a crucial step in all PCR applications to ensure specific and efficient amplification of target sequences. Despite the availability of numerous online and commercial bioinformatics tools, primer design remains inconvenient and impractical for routine use.

The evolving applications of PCR have further emphasized the need for advanced tools capable of addressing diverse requirements. These include quantitative fluorescent PCR, multiplex PCR with multiple primer combinations, discovery and amplification of simple sequence repeats for diagnostic markers,^6^ and the design of primers and probes (e.g., dual-labelled oligonucleotide probes, TaqMan, or molecular beacons). TaqMan and molecular beacon assays both use a reporter and quencher dye attached to the probe, which then hybridize to the target sequence during amplification. Developing such tools is essential for supporting the growing range of PCR-based applications.

PCR is a simple and rapid method for analyzing nucleotide polymorphisms and detecting gene variations, widely adopted across fields such as basic research, agriculture, and medicine. Among the various PCR methods, allele-specific PCR (AS-PCR) is notable for its ability to selectively amplify specific alleles through competitive reactions with allele-specific primers (ASPs).^7^^,^^8^ One prominent AS-PCR variant, Kompetitive allele-specific PCR (KASP), was specifically developed for fluorescence-based detection of amplification results. These techniques allow for the analysis of nearly any allelic variation, often using one universal primer (UP) in combination with multiple ASPs. The versatility and precision of these methods make PCR indispensable in modern science and technology.

The main purpose of this study was to present and validate a universal, practical, and user-friendly web-based platform that integrates multiple tools for primer design, genotyping assay development, repeat sequence analysis, and synthetic biology applications to support a broad range of molecular biology workflows. These include PCR-based workflows, LAMP,^5^ KASP genotyping assay development, oligonucleotide analysis and design, and accurate *de novo* identification, classification, and visualization of repetitive sequences.^9^ The design parameters used in the platform were grounded in experimental data to ensure efficient PCR performance, and were translated into algorithms capable of generating optimal primer-pair combinations for amplification. By implementing novel algorithmic approaches, this platform enables high-throughput analysis, while maintaining accuracy and reliability across a wide range of applications. Performance evaluations have demonstrated that the platform achieves superior sensitivity and specificity compared to existing solutions, with significantly reduced computational overhead, making it an efficient and reliable tool for researchers.

## Results

### Comparison with other software

**Figure 1:**
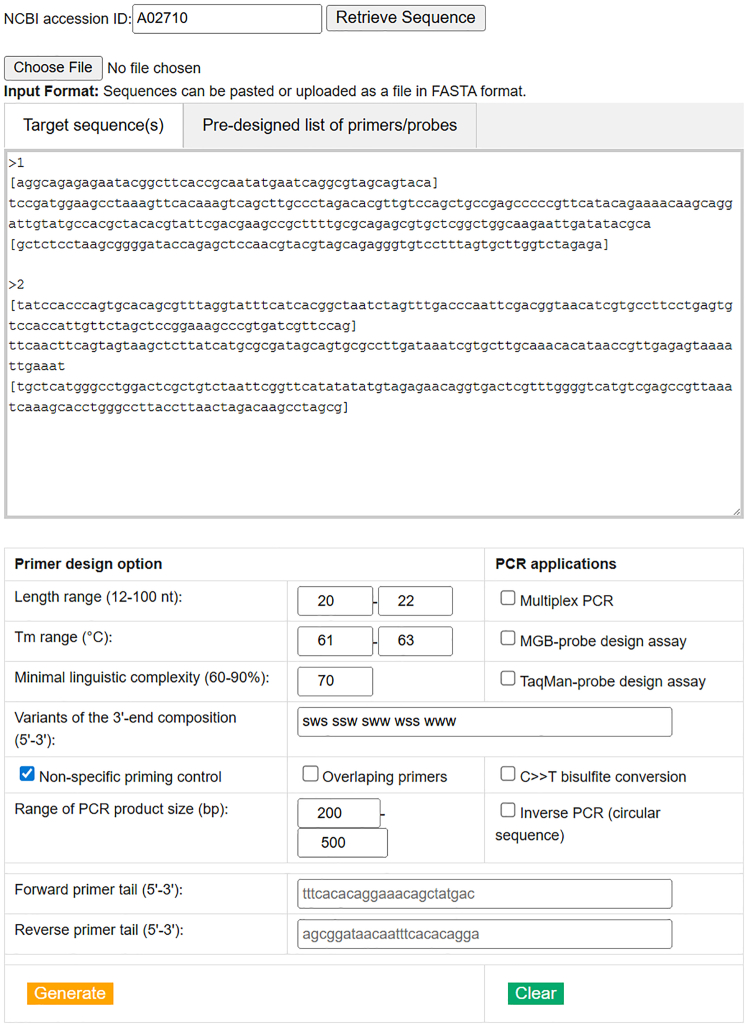
Web interface of “PCR, multiplex, and QF-PCR primer design and genotyping tool,” which provides advanced capabilities for designing primers across a wide range of PCR applications, including standard, inverse, multiplex, quantitative fluorescence (TaqMan or MGB dual-labeled fluorescent probe assay design), and bisulfite PCR It also supports the development and validation of primer sets for genotyping SNPs and InDels. Additionally, all individual tasks can be multiplexed efficiently for high-throughput analysis, such as fluorescence-based multiplex real-time qPCR assays. Sequences can be pasted or uploaded as a file in FASTA format, or retrieved NCBI accession (e.g., A02710) from the NCBI nucleotide database, or retrieved flanked sequence SNP sequences from the Ensembl database. One or more SNP or variant rs IDs (e.g., rs1357617) can be used to retrieve the surrounding sequences (±flank bases) for specific species names.

For comparison, we considered other web-based toolset software options to design primers or probes for a wide range of PCR applications, oligonucleotide analyses, and other tools for specific experiments. Integrated DNA Technologies (IDT) SciTools (http://eu.idtdna.com/scitools/) and New England Biolabs (NEB) Interactive Tools (https://www.neb.com/en/tools-and-resources/interactive-tools) are the most commonly used web-based toolset applications. The Sigma-Aldrich OligoArchitect online tool (https://www.oligoarchitect.com/) is a web-based toolset software primarily used for PCR and qPCR applications. Table 1 provides a comparison of the novel features of our web-based toolset software analysis (Figure 1). IDT SciTools and NEB Interactive Tools contain many specific tools, including unique ones. Our web-based toolset software contains both common and specific tools. Hence, it is possible to compare the overlapping features of these toolkits. IDT PrimerQuest generates high-quality PCR primers similar to our PCR and qPCR primer design tools. The primers did not form dimers, and the linguistic complexity (LC) values for the sequences were at least 68% (Tables S1 and S2). With our PCR tool, the user can control the lowest LC value and, if necessary, increase the value above the default value (LC = 75%). The LC value is an important parameter for characterizing primer or probe sequences, which determines the uniqueness and, indirectly, the specificity of the sequence. For the first time, we proposed the use of LC analysis to characterize the sequence of primers or probes in FastPCR software as a routine indicator.^1^ In our view, the majority of online PCR primer design tools do not account for the LC of primer sequences, relying primarily on melting temperature (Tm) as the main selection criterion. However, Tm alone is not a sufficient parameter to ensure efficient and specific PCR amplification.

**Table 1:** Comparison of the main web-based toolset software for advanced PCR applications and sequence analysis

Features	Web tools for PCR, qPCR, LAMP, electronic PCR, genotyping, analyzing primers, setting up reactions, and identification of repeats (https://primerdigital.com/tools/)	IDT SciTools web tools (http://eu.idtdna.com/scitools/)	NEB interactive tools (https://www.neb.com/en/tools-and-resources/interactive-tools)	Sigma-Aldrich OligoArchitect (https://www.oligoarchitect.com/)
**Input format**				

Text file	FASTA, GenBank (Accession/GI number), dbSNP ID (rs ID), space, and tab-delimited	FASTA, GenBank (accession/GI number), dbSNP ID (rs ID), gene symbol, or chromosomal range	FASTA, GenBank (accession/GI number)	GenBank (Accession/GI number)
Nucleotide type	standard bases (A, C, G, T, U, I), mixed, RNA and LNA	standard bases (A, C, G, T, U, I), RNA, mixed	standard bases (A, C, G, T)	Standard bases (A, C, G, T), LNA

**PCR and LAMP applications**				

Standard PCR	PCR, qPCR primer design tool	PrimerQuest	not supported	SYBR Green I assay
Multiplex PCR	PCR, qPCR primer design tool	not supported	not supported	not supported
Inverse PCR (circular)	supported	not supported	not supported	not supported
Bisulfite PCR	supported	not supported	not supported	not supported
Pre-designed primer/probe compatibility	supported	not supported	not supported	not supported
Linguistic complexity (LC) control	supported	not supported	not supported	not supported
Repeated sequences masked	with TotalRepeats	PrimerQuest not supported	not supported	not supported
Quantitative Fluorescent PCR	TaqMan, MGB	supported (Real-Time PCR, PrimerQuest)	not supported	Dual-labeled, molecular beacon, LightCycler and Scorpion probe
LAMP sets design	LAMP primer sets design tool (6 and 8 primers with loop primers sets)	not supported	NEB LAMP Primer Design Tool (loop primers not supported)	not supported
Site-directed mutagenesis	not supported	not supported	NEBaseChanger	not supported
Custom multiplex tiling PCR panel design	supported	not supported	not supported	not supported
DNA assembly or Gibson assembly	Gibson assembly primer design tool	not supported	NEBuilder Assembly Tool, NEBridge Golden Gate Assembly Tool	not supported
Limits	unlimited length and entries	<100	1	1

**Genotyping**				

Genotyping tool	KASP primers assay design tool	rhAmp Genotyping Assay Design Tool	not supported	not supported
Genetic variants	mono, bi- tri-, and tetra-allelic SNPs/InDels, multi-nucleotide variants, haplotypes	only biallelic SNPs are allowed	not supported	not supported

**Oligo, batch analysis and handling**				

Oligo analysis	PrimerAnalyser	OligoAnalyzer Tool, UNAFold	Tm calculator	not supported
Multiple primers analysis	PrimersList	Batch Analysis Tool	not supported	not supported
Dilution, conversions, and reaction mixer calculation	PrimerAnalyser, Universal dilution, and mixing two solutions calculators	Resuspension, dilution calculator	NEBioCalculator, thermostable ligase reaction temperature calculator, read coverage calculator	not supported
Limits	unlimited length and entries	<200	single sequence	not supported

**Tool for specific experiments**				

Restriction enzyme tools	not supported	not supported	double digest finder, enzyme finder, NEBcloner, NEBcloner	not supported
Experimental design	PCR reaction setup calculator, Oligo probe design for the Gator GeneSwift assay	codon optimization tool, gBlocks gene fragments entry	Glycan analyzer, NEBcloner, DNA sequences and maps tool, EnGen sgRNA template oligo designer, NEBNext custom RNA depletion design tool, PCR fidelity estimator	not supported
Gene regulation and RNAi	not supported	RNAi design tool	not supported	not supported
CRISPR genome editing	not supported	CRISPR HDR design tool	not supported	not supported
*de novo* identification, masking and clustering of repeated sequences	TotalRepeats	not supported	not supported	not supported
Virtual (*in silico*) or electronic PCR (ePCR)	*in silico* PCR tool	not supported	not supported	not supported
Required user authorization	not required	required	not required	not required

The capability of web-based tools to perform analyses for potential non-specific amplification within target sequences is essential, as repetitive elements constitute a substantial portion of eukaryotic genomes and often lead to non-specific amplification events. However, a limitation was identified in the PrimerQuest Tool (https://eu.idtdna.com/Primerquest/Home/), which does not provide support for efficient masking of repetitive sequences within target regions. In contrast, another tool offered by IDT for real-time PCR design (https://eu.idtdna.com/scitools/Applications/RealTimePCR/) incorporates repeat detection and actively excludes such regions from primer design, thereby reducing the risk of non-specific amplification.

The IDT Genotyping Assay Design Tool facilitates the design of single nucleotide polymorphism (SNP) genotyping assays using proprietary rhAmp SNP technology, which includes the rhAmp Genotyping Master Mix and rhAmp Reporter Mix. This approach enables the detection of biallelic SNPs, including those with minor allele frequencies greater than 1%. In contrast, our KASP primer assay design tool offers a universal solution for designing assays targeting any type of SNP or insertions/deletion (InDel), accommodating one to four allelic variants. It also supports the detection of multi-nucleotide variants and haplotypes. The polymorphic site can be positioned at the 3′ end of either primer, and the software either allows user specification or automatically identifies the most suitable location for optimal discrimination.

Oligo, batch analysis, and handling tools from the IDT and our study have common features as well as differences. For example, the IDT OligoAnalyzer incorporates mFold^10^ for the prediction of oligonucleotide secondary structures and provides the option to submit input sequences directly to the NCBI BLAST interface for short, nearly exact match searches. In contrast, our tool imposes no restriction on the number of primers analyzed simultaneously, and performs comprehensive assessments for self-dimer and cross-dimer formation across the entire input list. Primer and probe specificity is further evaluated using an *in silico* PCR tool. Moreover, our platform includes utilities for calculating stock concentrations and preparing dilution series for oligonucleotides. Aside from these distinctions, the remaining features of both tools are functionally equivalent.

Our LAMP primer design tool provides functions to design primer sets for LAMP applications. A similar application is available in NEB: the LAMP Primer Design Tool, which uses PrimerExplorer (https://primerexplorer.jp/e/).^5^^,^^11^ The differences between these tools are related to the specifications of the primer design algorithms and their evaluations, user friendliness, and additional features. In our tool, the user can more efficiently select any variants of the LAMP sets, including those for specific tasks, for example, for bisulfite conversion for both strands. In addition, a significant difference in our tool is the LAMP sets design with two additional “loop” primers that accelerates the reaction. Our tool includes a non-specific priming control to detect repeated sequences.

While several publicly available tools exist for PCR primer design, our web-based platform distinguishes itself as a fully integrated environment offering a comprehensive suite of features for a wide array of molecular biology applications. These include primer design for conventional and multiplex PCR, isothermal amplification methods such as LAMP, allele-specific PCR genotyping, oligonucleotide analysis, and precise *de novo* identification, classification, and visualization of repetitive sequences. The platform enables the simultaneous design of primers for multiple nucleic acid sequences and internal target regions, supporting various PCR strategies and their combinations. Its modular architecture ensures broad applicability, providing researchers with a flexible and efficient toolset suitable for diverse experimental workflows across molecular biology and related disciplines.

### Benchmarking and performance evaluation

To evaluate the performance of our platform in comparison with existing primer design tools, we conducted a comprehensive benchmarking analysis using standardized conditions across web-based applications: PrimerQuest (IDT) and our own platform available at https://primerdigital.com/tools/pcr.html. For this analysis, we used a synthetic DNA sequence of 6.7 kb bp in length, representing a challenging target composed of exonic regions, low-complexity fragments, and various classes of repetitive elements. Approximately 47% of the sequence consisted of repetitive sequences. This design allowed us to assess the performance of different primer design algorithms in identifying suitable primer pairs within problematic genomic regions, particularly those prone to secondary structure formation or non-specific binding.

The resulting primer sets were further evaluated using FastPCR software^1^ to assess the LC of individual primers, predict potential primer-dimer formations, and identify non-specific binding sites through *in silico* PCR analysis. The LC metric provides a quantitative measure of sequence uniqueness and hybridization specificity, which is particularly important for primers located within repetitive or homopolymeric regions. Using the IDT PrimerQuest tool, an initial set of 50 primer pairs was generated; following the removal of redundant sequences, 60 unique primers remained. Under identical design constraints (primer length: 19–22 nucleotides; Tm: 60°C–62°C), our platform produced 182 unique primers targeting the same genomic region, demonstrating a broader and more versatile design capacity.

The results revealed substantial differences between the two platforms.

PrimerQuest (IDT): LC values ranged from 31 to 95, with a mean LC of 79.7. Notably, seven primers exhibited very low complexity (LC < 70), including three polypurine-rich sequences such as 5′-GaGGaGGaGaGaGGaGaaGaa, 5′-caaaGaGGaGaGGaGGaGaaaG, and 5′-GaGGaaacaaaGaaGaGGaGGa. Such low-complexity primers are generally unsuitable for PCR due to their high propensity for non-specific binding and self-dimer formation. The average GC content was 47.5%, and the mean Tm was 60.1°C. *In silico* PCR analysis revealed up to 63 unintended binding sites in repetitive genomic regions for nine of the designed primers, indicating insufficient masking or recognition of genomic repeats during design.

Our PCR Tool: LC values ranged from 76 to 100, with a higher mean LC of 86.0. No primers exhibited complementarity to known genomic repeats, microsatellite motifs, or polypurine tracts. The average GC content was 49.2%, and the mean Tm was 59.9°C. All primers exclusively amplified their intended targets with no off-target matches in repetitive or low-complexity regions, as confirmed by *in silico* PCR. Performance-wise, our tool designed 295 PCR primer sets in less than one second, compared to a time-to-result of under a few seconds for 50 sets in PrimerQuest. Median LC values were notably higher with our platform (86 vs. 80), reducing the likelihood of hybridization with problematic genomic regions. Collectively, these findings demonstrate that our tool enables more robust and specific primer design in repeat-rich genomic contexts, achieving greater sequence complexity, improved specificity, and superior computational efficiency relative to existing tools. A detailed comparative dataset, including primer-level metrics and *in silico* PCR validation, is provided in Tables S1 and S2.

### Laboratory validation: Design of multiplex tiling PCR pools

A total of 45 primer pairs were designed to tile across the entire reference SARS-CoV-2 genome (NCBI accession: NC_045512). Each amplicon spans approximately 1.2 kb, with overlapping regions of 500–1000 bp to ensure complete genome coverage and redundancy (Table S3). Primer pairs were optimized for compatibility with standard PCR conditions and designed to support multiplex PCR by combining primers from distinct pools without cross-reactivity. This modular design enables flexible assembly of primer sets tailored to a wide range of applications, including both qualitative and quantitative detection, as well as targeted sequencing on various platforms.

**Figure 2:**
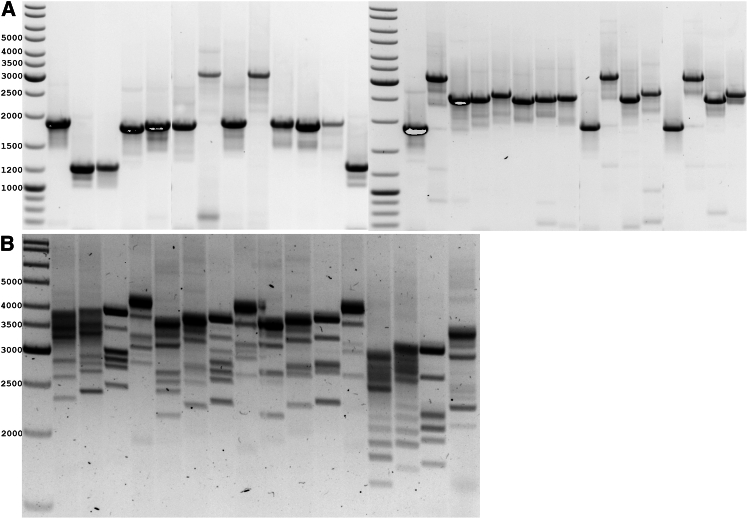
Validation of individual PCR pairs and multiplex PCR to analyses the SARS-CoV-2 genome (A) Individual analysis of combinations of PCR pairs from different parts of the viral genome. (B) Multiplex PCR analysis to cover the entire SARS-CoV-2 genome using different primer pair combinations from different parts of the virus genome. The PCR fragment size was determined using the GeneRuler DNA Ladder Mix (Thermo Fisher Scientific).

Multiplex tiling PCR pools with 1.2 kb amplicons were constructed and can be adapted to produce longer or alternative-sized fragments by integrating compatible primers from multiple pools. The performance of each primer pair was validated using conventional PCR with Phusion Hot Start II DNA Polymerase (Thermo Fisher Scientific) under optimized conditions to confirm amplification efficiency and specificity.^12^ During validation, the quantitative amplification efficiency of selected genomic regions across the SARS-CoV-2 genome was assessed using intact viral sequences. Multiplex PCR was performed using primer sets composed of several primer pairs (up to 12 pairs) targeting distinct regions from the 3′ and 5′ ends of the genome. Amplified fragment identity was confirmed to verify sequence integrity. The protocol enables whole-genome amplification of SARS-CoV-2 using tiled amplicons of up to 4.8 kb, which is particularly suitable for low-titter clinical samples (Figure 2). Laboratory validation demonstrated efficient and consistent amplification across all primer sets, with robust performance of the multiplexed tiling PCR pools throughout the entire SARS-CoV-2 genome. This genome-wide coverage was reproducibly achieved even in samples with low viral loads, highlighting the high sensitivity and efficacy of the developed multiplex PCR approach. Quantitative assessment revealed high and uniform amplification yields across targeted genomic regions, with minimal inter-primer variability. Specificity testing confirmed that all primer sets selectively amplified the intended SARS-CoV-2 targets without significant off-target amplification or cross-reactivity. The developed multiplex PCR system supports flexible primer-pool configurations tailored to specific sequencing or diagnostic applications, allowing rapid adaptation for use in genomic surveillance, variant detection, and research workflows.

## Discussion

We present a web-based platform that provides an integrated, accessible, and functional environment for primer design, genotyping assay development, sequence analysis, and repeat identification. It encompasses a wide range of PCR-related applications, including standard and multiplex PCR, bisulfide-converted templates, LAMP and KASP assays, *in silico* PCR simulations, Gibson assembly primer design, and *de novo* detection and classification of genomic repeats. By consolidating these functionalities into a single-client-side application, the platform meets the increasing demand for rapid, high-throughput, and user-friendly tools in modern molecular biology.

A web-based platform revealed superior computational efficiency, improved primer specificity, and reliable handling of complex sequence features such as low-complexity regions and interspersed repeats. These results underscore the platform’s practical applicability and effectiveness in both diagnostic and research settings, particularly in scenarios requiring high sensitivity, target specificity, and genome-wide coverage.

Despite these strengths, this study had several limitations that must be acknowledged. As a purely browser-based system, performance may be limited when analyzing large datasets, especially when conducting repeat analysis of eukaryotic genomes exceeding 50 Mb or when working on machines with limited memory. Additionally, in complex multiplex primer design scenarios, it may not always be possible to identify optimal primer sets within fixed, user-defined constraints. In such cases, the algorithm does not impose control over each design outcome or apply alternative parameters for automated optimization. Instead, it leaves the decision to the user, allowing for manual interpretation and assessment of primer suitability based on the specific experimental context. The quality of input sequences inherently influences primer design outcomes. The presence of ambiguous nucleotides or extensive low-complexity regions can compromise primer quality, often resulting in suboptimal or non-specific primer candidates. The algorithm identifies such cases using a LC score of zero, which suggests that the resulting primers may require user review or repetitive content masking. Furthermore, while the platform supports extensive parameter customization, inappropriate settings such as overly broad Tm ranges or minimal specificity thresholds may result in non-functional or non-specific primer pairs/sets. To mitigate such risks, the algorithm incorporates default parameter presets and built-in warning mechanisms to guide users during the design process. Nevertheless, expert oversight remains essential, particularly when using advanced or non-standard configurations, to ensure the accuracy and applicability of the resulting primers. Finally, *in silico* PCR and non-specific binding analyses rely on publicly available reference genome databases. Currently, chromosome-scale analysis of large eukaryotic genomes requires the uploading of individual chromosome sequences, which can restrict the automation of high-throughput workflows. While these limitations do not detract from the platform’s overall utility, they highlight areas for future enhancement. Overall, the platform offers a high-performance, flexible framework for a wide array of nucleic acid analysis tasks with a design philosophy focused on accuracy, transparency, and usability.

## Materials and methods

The web tools, accessible at https://primerdigital.com/tools/, were implemented in JavaScript to ensure universal compatibility across all operating systems. These tools accept input as either a single DNA or RNA sequence, multiple sequences in FASTA format, or sequences retrieved from the GenBank database, as well as flanking SNP sequences of one or more SNP/variant rs IDs, which provide flexibility for a wide range of molecular biology applications.

### PCR primer design generalities

PCR primer design is a key step for successful PCR experiments. PCR primers typically range from 18 to 35 bases in length and should be designed to exhibit complete sequence complementarity with the target fragment intended for amplification. The following parameters were controlled for primer length, Tm, minimum LC (%), and PCR product size (bp). These parameters are sufficient to achieve efficient and rapid results. Additionally, the user has the option to modify the parameters for identifying repeats in the target sequence using non-specific priming control and selecting overlapping primers if further primers need to be designed. Primer Tm was calculated using the nearest-neighbor thermodynamic parameters. The other main parameters used for primer selection were the general nucleotide structure of the primer, such as LC (nucleotide arrangement and composition), self-complementarity test, and secondary (non-specific) binding analysis.

All PCR primer (probe)-design parameters were considered flexible and amenable to modification based on the specifications of the analyzed sequence and the task to be performed. The primer pairs were analyzed for cross-hybridization. The specificity of both primers was determined based on their similarity in Tm values. Utilization of designed primers with balanced Tm (within 1–3°C of each other) is considered favorable, but not obligatory.

Several web-based primer- and probe-design software packages have been developed. Most of these are dedicated to the design of PCR primers or probes, and are based on Primer3 code.^2^^,^^3^ Our web tools are based on unique algorithms and efficient codes, providing a more flexible approach for designing primers for various applications. The authors evaluated whether the primer or probe possessed secondary binding sites in the input sequences that may have resulted in an additional PCR product. The proposed primer pairs differed from one another, with selection of the optimal pairs from all possible combinations. Users can modify the PCR amplicon size or design primer pairs for the entire sequence without specifying the parameters by using default or pre-designed parameters. Users can also specify multiple locations for both the forward and reverse primers by using “[ ]” in each sequence. The software allows multiple and independent locations for both forward- and reverse-primer design within each sequence, whereas PCR design is performed independently for different targets. The results obtained represent the suggested primers and primer pairs in tabulated format. The tabulated spreadsheets presented the following properties: automatically generated primer name, primer sequence, sequence location, direction, length, Tm, GC content, and LC (%).^1^ Annealing temperature (Ta) and PCR product size were determined for the compatible primer pairs.

### Melting temperature of oligonucleotides

The Tm of short oligonucleotides with standard or mixed nucleotide combinations was calculated using default parameters and nearest-neighbor thermodynamic values.^13^^,^^14^^,^^15^^,^^16^^,^^17^ The Tm of the mixed bases was calculated by averaging the nearest-neighbor thermodynamic parameters (enthalpy and entropy values) at each mixed site. Correspondingly, the extinction coefficient was estimated by averaging the nearest-neighbor values at the mixed sites.

### Linguistic complexity of sequence calculation

The sequence analysis complexity calculation method can be used to identify conserved regions between compared sequences and detect low-complexity regions, including simple sequence repeats, complex tandem repeats, or satellite repeats. LC measurements were conducted using the alphabet capacity k-mer method along the entire sequence length; they were calculated as the sum of all observed ranges from 1- to L-size words in the sequence, divided by the sum of the expected value for this sequence length. Complexity values were subsequently converted to percentage values, with 100% representing the maximum level of sequence complexity.^18^

### Oligonucleotide dimer formation

Web-based tools eliminate intra- and inter-primer dimers before generating the primer lists and candidate pairs. For optimal PCR efficiency, it is crucial to avoid the formation of stable and inhibitory dimers, particularly complementarity at the 3′ ends of the primers, from which the polymerase will extend. Stable primer-dimer formation significantly inhibits PCRs because the formed dimers are highly efficient in amplifying and thus compete with the target in PCRs. Primer dimers were predicted based on the analysis of the non-gap local alignment and stability of the 3′ end and central part of the primers. Primers were rejected if they exhibited the potential to form stable dimers of at least five bases at the 3′ end or seven bases in the middle. These conditions strictly followed the control dimers. Although there is a risk that some suitable primers will be eliminated, this is justified because reducing the cost of the reaction and the time required are of greater importance. Web-based tools calculate the Tm of primer dimers with mismatches for pure and mixed bases using the averaged nearest-neighbor thermodynamic parameters provided for DNA/DNA duplexes.

### Non-specific binding test (alternative amplification)

Oligonucleotide specificity is a crucial factor for the efficacy of PCR. Optimal primers should hybridize exclusively to the target sequence, particularly when complex genomic DNA is used as a template. Amplification issues may arise from the designed primer annealing of repeat sequences (retrotransposons, SINE, LINE, or tandem repeats). Alternative product amplification can also occur when primer sequences are complementary to the inverted repeats, resulting in the production of multiple bands. Inter-repeat amplification polymorphisms have exploited highly abundant dispersed repeats, such as the LTRs of retrotransposons and SINE-like sequences.^19^^,^^20^ The use of primer sequences complementary to any of these repeats may result in the production of numerous non-specific bands during single-primer amplification. The association of these sequences facilitated the amplification of a series of bands (genome fingerprints) using primers homologous to these high copy number repeats. The bands generated were highly informative as genetic markers. High copy number repeats can compromise the performance of specific PCR experiments, and are, therefore, best avoided unless the experimental design specifically aims to target them. A homology search of the primer sequence using, for instance, our “*in silico* PCR tool” against all publicly available genomic sequences helps determine whether the primer is likely to interact with interspersed repeats.^21^ By default, most PCR tools perform non-specific binding tests for each primer, although this function is typically disabled by the user. A secondary non-specific binding test based on the TotalRepeats tool identifies a wide range of repetitive elements, including telomeric and microsatellite repeats, ensuring a comprehensive repeat analysis across diverse genome types.

### С>>T bisulfite conversion

Bisulfite genomic modification is a central technique in DNA methylation analysis, and is widely used in both research and clinical settings.^22^ During this process, sodium bisulfite treatment converts unmethylated cytosines into uracil, which is then amplified as thymine. In contrast, levels of methylated cytosine (5-methylcytosine) remained unaltered. Consequently, when bisulfite-converted DNA is sequenced, unmethylated cytosines appear as thymines, whereas methylated cytosines remain as cytosines. By comparing the bisulfite-converted DNA sequence to the untreated DNA sequence, researchers can accurately determine the DNA methylation pattern. A variety of primer design tools, including PCR, qPCR, *in silico* PCR, LAMP, and KASP, facilitate the design of primers specifically tailored to bisulfite-converted DNA. These primers were designed to target both strands in a manner that ensures that only non-methylated cytosines (i.e., those not followed by guanine in CpG contexts) are converted to thymine, thus preserving the methylation information at CpG sites for precise downstream analysis.

### Hydrolysis probe design

PCR tools support the design of highly efficient hydrolysis dual-labelled fluorescent probes for TaqMan and minor groove binder (MGB) assays,^23^ which are essential for accurate and specific DNA quantification in quantitative fluorescent PCR applications. Both TaqMan and MGB probe assays employ a dual-dye system, with a reporter dye and a quencher dye attached to the probe. The oligonucleotide probe hybridizes with the target sequence, and fluorescence is generated when the proximity between the reporter and quencher is disrupted. Fluorescence is released through the 5′-to 3′- exonuclease activity of Taq polymerase, which cleaves the probe during DNA synthesis and separates reporter and quencher dyes. Both systems provide an indirect measure of hybridization in which the fluorescence signal is proportional to the target amplification. Probes should not contain a guanine residue at the 5′ end. TaqMan probes must be designed with a GC content of 45–65%, high complexity, no dimers with primers, a high Tm (68°C–72°C), and a probe length of 15–30 bp. The probe Tm should be 8°C–10°C higher than the primers. The 3′ MGB moiety non-covalently binds to the minor groove of the target, stabilizing the probe-target duplex and increasing its Tm.^23^ This stability allows for shorter probe designs that enhance specificity. MGB probes must be designed with high complexity, no dimers with primers, a probe length of 13–30 bp, with the probe Tm similar to the primer Tm. Both TaqMan and MGB probes offer a reliable and highly specific method for detecting target sequences, with signal generation tied directly to the separation of reporter and quencher dyes. This ensures the precise quantification of DNA in quantitative fluorescent PCR applications.

### Multiplex primer design

Multiplex PCR enables the simultaneous amplification of multiple DNA targets in a single reaction, thereby increasing efficiency and reducing the number of required reactions. Designing multiplex PCR assays to address a large number of target sequences is complex because of the combination of compatible primer pairs associated with the analysis of potential primer cross-interactions.

Primers must bind efficiently to their targets, and conditions such as Ta must be optimized to ensure uniform amplification. Our tool automates multiplex primer design, adjusts parameters to maximize compatibility, and minimizes non-specific amplification. The software uses an original algorithm that does not require complex or time-consuming computation. In all PCR applications that require the design of compatible primer pairs, it is possible to use a list of primers or probes as prerequisites for the compatibility of new sets. Thus, the designed PCR assays were compatible with the predesigned primer and probe list. The algorithm for designing multiplex PCR assays used a similar approach. A list of primers obtained by collecting all the primers from the previous sequences was used for each target sequence. Thus, for each sequence, a list of predesigned primers and probes was used to obtain compatible primers and probes. This approach significantly accelerates the selection of compatible PCR assays for any number of target sequences. This algorithm is effective because the number of potential primer variants is sufficient for each target sequence. In situations in which the number of potential primer variants is extremely limited, the design of compatible multiplex PCR assays for a particular sequence may be limited. In this case, the algorithm analyses the number of potential primer variants in specific sequences in advance, and uses these target sequences at the beginning of the list of sequences to be analyzed. Therefore, the target sequences will be at the beginning of the list of sequences to be analyzed, and the resulting potential primer variants will be included in the list of pre-designed primers. For downstream sequences, the designed potential primer variants were analyzed for compatibility with this list.

Ta is essential for designing compatible primers with similar Tm values, as the amplification conditions for multiplex PCR assays should be optimal for all amplicons. Ta depends on the nucleotide composition, amplified fragment length, and Tm of the primers used. The shorter PCR product was amplified efficiently at lower temperatures (e.g., 100 bp at 55°C), whereas the longer fragments required higher temperatures (e.g., 1000 bp at >60°C). Therefore, size compatibility of PCR products should be considered, as a high variation in size may reduce amplification for certain types of amplicons. The PCR product sizes should be close and compatible for efficient amplification under the same conditions.

The Tm compatibility of the primers was determined using a simple method. Thus, in the options for primer design, it is necessary to specify the narrow range of Tm at which the primers should be matched. For example, for most PCR tasks, the Tm range in which primers are preferably designed is between 60°C and 62°C, calculated by considering the conditions of the reaction mixture composition (50 mM KCl, 1.5 mM Mg^2+^, and 0.8 mM dNTP). If other temperature conditions are required for PCR primers, a narrow Tm range for all designed primers should also be used. The PCR tool allows users to refine designs by selecting pre-designed primers or adjusting conditions such as product-size differences. This provided a comprehensive analysis of primer compatibility, including potential dimers, cross-hybridization, and alternative primer pairs. To prevent non-specific amplification, the software filters the primers to avoid repeated or problematic motifs.

Additionally, the tool facilitates the design of thermodynamically and sequence-compatible primer pairs for a wide range of molecular applications. This includes conventional PCR, allele-specific and SNP genotyping assays, as well as the construction of multiplex reaction panels incorporating dual-labelled fluorescent hydrolysis probes, such as those employed in TaqMan or MGB assays. The primer design algorithm accounts for cross-reactivity, Tm compatibility, and optimal amplicon characteristics to ensure assay specificity and efficiency across multiple targets.

### Loop-mediated isothermal amplification primer design tool

In its original design, LAMP used four synthetic primers derived from six target regions: F3, F2, F1c, B1c, B2, and B3.^5^ The sequences from F1c and F2 were merged into one primer (FIP), and those from B1c and B2 were merged into another primer (BIP). In the revised LAMP method, two additional primers (loop primers) were introduced, yielding six primers that recognized eight distinct regions of the target DNA, thus ensuring highly specific amplification. Among these six primers, four “core” primers drive the amplification, and two “loop” primers accelerate the reaction. The core primers produced DNA with two inverted self-complementary regions, forming a self-hybridizing loop at both ends of the target sequence. This arrangement provides multiple priming sites for strand displacement of *Bst* DNA polymerase, leading to rapid exponential amplification. This process generates the concatemers mentioned above, and rapidly produces long chains of short target sequences. The resulting amplification products, extending over 20 kb, consisted of multiple repeats of a short (200–350 bp) target sequence linked by single-stranded loop regions to the extended concatemers.

The steps involved in designing the LAMP primer sets included masking repeated sequences within the target regions; independently creating lists for each primer type (F/B1, F/B2, F/B3, and LPF/B) specific to the target area; assembling compatible single primers into comprehensive LAMP primer sets for both directions; and integrating these sets into the LAMP assay, considering primer spacing and constraints on the F2-B2 amplicon length set by the user. Our tool eliminates intra- and inter-primer dimers from the primer set. By default, repeated regions within the target sequence were excluded from analysis. Additionally, the design of the LAMP assay allowed for bisulfite conversion in both strands.

### KASP primers assay design tool

The KASP design tool performs primer assays for SNP, InDel multi-nucleotide variants, and haplotype polymorphisms as applied to KASP-like technologies. KASP (LGC Biosearch Technologies, Teddington, UK) and PCR Allele Competitive Extension (Integrated DNA Technologies, Inc.) are AS-PCR technologies used to detect sequence variability using just one nucleotide base at a particular locus. Various methods have been developed for this purpose. These methods can be used to analyze almost any allelic variation, and usually represent PCR with one UP and several ASPs. Thus, in AS-PCR, the UP is paired with an ASP or several ASPs. UP perfectly anneals to all allelic variants, whereas ASP perfectly anneals to one allele without mismatches. For other allelic variants, ASP anneals with a mismatch at the 3′ terminal base or in a position close to the 3′-terminus. The design of this test allows for the preferential amplification of each allele with the participation of a specific ASP.^7^^,^^8^^,^^24^

For all polymorphisms considered in this study, the program produced a thermodynamically optimal combination of ASPs and UP for single-plex or multiplex assays. Primers for KASP were selected based on the same general principles as those used for standard PCR, provided that a multiplexing option was considered. The thermodynamic stability of possible hairpins should always be evaluated for the 3′ termini of the primers; the tool performs these calculations. If desired, the program could perform a thermodynamic stability analysis of the secondary structures in all primer sequences. The program automatically checked the primers used for the formation of the self-dimers and cross-dimers. The computing parameters were saved and reused for other calculations. The tool produces sets of primers capable of amplifying all alleles provided in the input data of the program. The output contained primer sets that were ranked automatically to simplify selection of the best set. This program can be used to plan primers to amplify PCR products of desired size. Alternatively, the tool may be requested to search for primers for diagnostic PCR, in which case, an entire input template will be searched to find the best primer landing sites. The program also automatically selected primers to avoid sequence repeats and stable motifs such as G-quadruplexes.

All computed results can be exported in tabulated formats. The following data describing the computed primers were exported to spreadsheets: primer name, sequence, target location, sequence direction, length, Tm, GC content, and LC. For example, for pairs of primers intended for use in one reaction, the program computes the Ta, PCR product size, and Tm of the PCR product. The extracted data included Ta's, identification of a common primer pair target, list of compatible primers, and PCR product sizes.

### Primer(s) analyses

Individual primers and sets of primers were evaluated using the PrimerAnalyser or PrimersList. Primer Tm values were calculated using default or other formulas for normal and degenerate nucleotide combinations, GC content, extinction coefficient, unit conversion (nmol per optical density [OD]), mass (μg per OD), molecular weight, and LC.^14^ Users can select either DNA or RNA primers with normal or degenerate oligonucleotides, or primers that can be modified with different labels (e.g., inosine, uridine, or fluorescent dyes). The tools report the nearest-neighbor thermodynamic parameters and simple non-thermodynamic Tm calculation formulas. For locked nucleic acid (LNA) modifications, four symbols were used: dA = E, dC = F, dG = J, and dT = L.^25^ Both tools perform analyses of type, which allow users to see the results immediately on the screen. They can also calculate the volume of solvent required to attain a specific concentration from a known mass (mg), OD, or number of moles of dry oligonucleotide. All primers were analyzed for intra- and inter-molecular interactions to form dimers. Primer(s) can efficiently hybridize using the 5′ end or middle of the sequences. Such dimers are not efficiently extensible by DNA polymerase; however, they affect the primer concentration and can strongly inhibit DNA polymerase activity during PCR. Higher concentrations of double-stranded DNA strongly inhibited DNA polymerases.

### *In silico* (virtual) PCR

Hybridization of primers or probes to target annealing sites is the sole method available for PCR product prediction.^21^ Primer or probe sequences can potentially bind to numerous sequences in the template; however, only attachment sites with minimal mismatches (one or two, depending on their location at the 3′ end of the primer) may enable the polymerase to perform strand elongation. The terminal 12 bases at the 3′ end of the primers were critical for overall primer stability on the template and for the initiation of DNA extension. Individual mismatches in the terminal 12 bases at the 3′ end of the primer can diminish primer binding and PCR efficiency, with the effect being more pronounced near the 3′ end. *In silico* PCR tools facilitate concurrent testing of individual or multiple primer or probe sequences designed for multiplex target sequences. These tools execute a rapid, gapless alignment to assess the complementarity of the primers with the target sequences, and the parameters can be adjusted to accommodate varying degrees of mismatch at the 3′ end of the primers. This tool is also capable of processing sequences with no complement to the 5′ or 3′ tails of the target sequence. Rapid alignment for the detection of primer sequence locations on the target sequence was conducted by analyzing both DNA strands using a hash index of 12-mers (allowing up to one mismatch) and by calculating the local similarity for the entire primer sequence. Potential primer-binding sites and PCR amplicons can be identified for linear and circular templates by using standard or inverted PCR and multiplex PCR. This *in silico* tool presents an efficient method for rapidly analyzing primer or probe sequences against target sequences to determine primer location, orientation, binding efficiency, and Tm.

Additionally, the tool supports microRNA (miRNA) target site screening within input sequences.^26^ To ensure high specificity, users are required to define the maximum number of tolerated mismatches, which should not exceed three. This constraint enables the accurate prediction of potential miRNA-binding sites while minimizing false positives. Furthermore, the utility of the application is substantially extended by its capability to conduct genome-wide or chromosome-specific searches for sequences complementary to CRISPR-Cas guide RNAs. This feature is essential for optimizing genome editing specificity, as it allows for the systematic evaluation of potential off-target sites. By enabling precise alignment and mismatch parameterization, the tool assists researchers in refining guide-RNA designs to reduce unintended genomic modifications.^27^

### Gibson assembly primer design tool

Gibson Cloning is a technique for assembling DNA constructs that allows multiple linear segments to be joined either into a single large linear segment or, if the segments contain the appropriate components and overlap, into an intact plasmid.^28^ An isothermal, single-reaction method for the assembly of multiple overlapping DNA molecules is achieved by the concerted action of a 5′-exonuclease, DNA polymerase, and DNA ligase. This assembly method can be used to seamlessly construct synthetic and natural genes, genetic pathways, and entire genomes, and could be a useful molecular engineering tool. For successful assembly, DNA fragments must typically share at least 20–40 bp homology with adjacent segments, ensuring a Tm of >50°C in the overlapping region. The user must arrange the DNA fragments in the desired assembly order. While not mandatory, including the vector sequence at both the beginning and end of the list can help streamline the process. The software-designed primers at the ends of each sequence are effective for PCR under long fragment amplification conditions. For example, the range of Tm in which primers are preferably designed is between 60°C and 62°C, calculated considering the conditions of the reaction mixture composition (50 mM KCl, 1.5 mM Mg^2+^, 0.8 mM dNTP). Users can modify the parameters to select primers for any other condition. Each primer contained a non-complementary tail complementary to the neighboring sequence. When a non-complementary tail was formed, the primer sequence from the neighboring sequence was used. The absence of intramolecular and intermolecular dimers is necessary for the compatibility of a pair of primers. To assemble DNA constructs into a vector, primers for sequences bordering the vector contained a non-complementary tail from the vector sequence. To amplify the vector itself, the software selects a pair of primers for inverted PCR to amplify the vector sequence.

### *De novo* identification and visualization of interspersed and tandem repeats

A genomic repeat analysis tool (TotalRepeats) was designed to efficiently identify and cluster any type of repetitive sequence in the target sequence (genome). The algorithm can identify various types of repeated sequences, including perfect and imperfect microsatellite repeats and short tandem repeats belonging to a wide range of higher-order repeat structures of large satellite sequences and telomeres.^9^ This tool provides a rapid and automated method for the repeated sequence identification, classification, and comprehensive analysis of *de novo* repeats associated with genes, their families, and mobile elements. *De novo* identification of repetitive sequences is based on the transformation of k-mer frequencies to accurately determine the repeat boundaries. Following the identification of all repeat blocks and the definition of their boundaries, the software arranges the repeat blocks in descending order of length to facilitate efficient clustering and homology detection. Subsequently, it evaluates the homology of each repeat against all others using a novel algorithm specifically designed for high-speed approximate string matching based on the k-mer vector algorithm. TotalRepeats clusters the resulting repeat blocks of the target sequence(s).

The output includes masked repetitive sequences and graphical files. The results were stored in a tab-delimited text file that provided a sequential list of homologous sequence clusters, including start and end coordinates, orientation, and sequences. In the visualization of the results, forward-oriented sequences are depicted in blue, whereas reverse-oriented sequences are depicted in red in the output PNG graphic file.

## Data availability

The web-based tools described in this study are freely accessible at https://primerdigital.com/tools/ and are intended for academic and research use without any registration or licensing requirements. The platform is implemented entirely in client-side JavaScript, ensuring that all the algorithmic code is delivered directly to the user’s browser and can be locally inspected. All computations and data handling are performed locally on the user’s device, no data are transmitted to external servers, and no user information is collected or stored. The platform was maintained by the author for non-commercial and scientific purposes and was not affiliated with any commercial entity. The selected source code modules are publicly available on GitHub: https://github.com/rkalendar/PCRtools.

## Acknowledgments

This study was funded by the Committee of Science of the Ministry of Science and Higher Education of the Republic of Kazakhstan (grant nos. AP23483529, BR24993023, BR24992881, BR27199879, and BR24992841).

## Author contributions

R.K. proposed the original ideas, conceived the study, performed programming, and wrote the manuscript.

## Declaration of interests

The author declares no financial or commercial conflict of interest. The author maintains the website (https://primerdigital.com/tools/) solely for academic, research, and educational use. The tools are provided free of charge, without registration, and with no commercial licensing. The author received no financial compensation from hosting or maintaining the platform.
